# Co-Existing Charcot-Marie-Tooth Disease Type II and Parkinson’s Disease Linked to a Novel DNAjB2 Pathogenic Variant

**DOI:** 10.1177/11795735261428314

**Published:** 2026-03-04

**Authors:** Alexandru N. Lerint, Johanna S. Canenguez Benitez, Vijaya Lakshmi Valaparla, Elena Shanina, Laura J. Wu

**Affiliations:** 1 12338 Department of Neurology, University of Texas Medical Branch, Galveston, TX, USA

**Keywords:** DNAjB2 gene, familial Parkinson’s disease, hereditary neuropathies, dual phenotypes, compound heterozygous variant

## Abstract

**Background:**

The DNAjB2 gene encodes a co-chaperone protein that interacts with the heat shock protein (HSP) family to maintain protein quality control and preserve neuronal integrity. Variants in this gene have been associated with the axonal form of Charcot-Marie-Tooth Disease (CMT2). Recent literature has also suggested an association between DNAjB2 variants and neurodegenerative disorders such as Parkinson’s disease (PD).

**Design/Methods:**

Case Report.

**Case Description:**

We present a 36-year-old female patient initially diagnosed with CMT2 at the age of 28, who later developed symptoms of PD in her fourth decade. Genetic test revealed compound heterozygous pathogenic variants in DNAjB2 (c.352+1 G>A and c.175+2T>A).

**Conclusion:**

To our knowledge, this is the first case report describing the dual phenotype of CMT2 and young-onset PD linked to compound heterozygosity in DNAjB2. The dual dysfunction of axonal degeneration and dopaminergic neuron loss suggests that DNAjB2 plays a pivotal role in maintaining proteostasis in both the peripheral and central nervous systems.

## Plain Language Summary

This case report discusses a rare case of a 36-year-old female diagnosed with Charcot-Marie-Tooth (CMT) type 2 disease who later developed Parkinson's disease (PD). A few other family members were diagnosed with CMT and PD. Her genetic testing revealed a mutation in the DNAjB2 gene. The DANjB2 gene is essential for maintaining healthy proteins in both the central and peripheral nervous systems. Dysfunction of this gene leads to neurodegeneration and the development of CMT (peripheral) and PD (central). This gene could be a potential therapeutic target for such overlapping clinical phenotypes.

## Introduction

Charcot-Marie-Tooth (CMT) disease refers to a group of genetically heterogeneous disorders with autosomal dominant, autosomal recessive, and X-linked inheritance patterns. Variants in the same gene can lead to different CMT phenotypes (allelic heterogeneity), while variants in other genes can cause similar CMT presentations (locus heterogeneity). To date, more than 100 genes have been identified as contributors to CMT including DNAjB2.^1^

DNAjB2, a member of the DNAj protein family (also known as heat shock protein 40, HSP40), is located on chromosome 2 and is predominantly expressed in the brain, spinal cord, and nerves.^2^ This gene encodes a co-chaperone protein essential for protein quality control and the cellular stress response through interactions with the HSP family. The DNAjB2 protein facilitates the refolding or degradation of misfolded proteins, a process critical for neuronal integrity. Disruptions in DNAjB2 can lead to the toxic accumulation of misfolded proteins and impaired cellular degradation, affecting both the central and peripheral nervous systems.^2-4^ Studies have identified multiple distinct variants in the DNAjB2 gene that cause autosomal recessive axonal CMT (CMT2).^3^ These include missense, nonsense, frame shift, large deletion, and splice-site variants, resulting in the loss of protein function (Table 1).^2,3,5-11^

**Table 1.** DNAjB2 Protein Structure Highlighting the Locations and Types of the Various Variants Reported to Cause Autosomal Recessive Axonal CMT2. Variants Include Missense, Nonsense, Splice-Site, and Frameshift Variants Distributed Across the J-Domain, G/F-Rich Region, and C-Terminal Domain Ref. 2

Variant	Type	Phenotype	Reference(s)
c.352+1G>A	Splice-site	CMT2, YOPD	Frasquet et al.^7,8^
			Teive et al.^11^
c.125C>A	base substitution at coding-sequence nucleotide 125	YOPD	Frasquet et al.^8^
c.229+1G>A	Splice-site	CMT2	Gess et al.^9^
c.620-1G>A	Splice-site	CMT2	Lazzarini et al.^10^
c.310delC	Frameshift	CMT2	Lazzarini et al.^10^
3.8 kb deletion	Large deletion	CMT2 + Parkinsonism/SMA	Sanchez et al.^12^
c.14A>G (p.Y5C)	Missense	Distal hereditary motor neuropathy (dHMN)	Sarparanta et al.;
			Saveri et al.^2,3^
g.219277938_219281781del	Likely null	dHMN, YOPD	Sanchez et al.^12^
c.145delG	Frameshift	CMT2, YOPD, hearing loss	Saveri et al.^3^

YOPD: Young-onset Parkinson’s Disease; CMT2: Charcot-Marie-Tooth Type 2; SMA: Spinal Muscular Atrophy

Recent research on the DNAjB gene family has identified its association with neurodegenerative diseases through multiple mechanisms. DNAjB2 directly interacts with HSP70, with its J domain and ubiquitin-interacting motif binding to ubiquitinated substrate proteins, facilitating their degradation. The dysfunction of this mechanism, influenced by the DNAjB2 gene, is linked to impaired clearance of neurodegenerative products, such as alpha-synuclein, the main pathognomonic protein associated with Parkinson’s disease, leading to its accumulation in the central nervous system.^4^ Huang et al. reported that epidermal growth factor receptor (EGFR) mediated phosphorylation of DNAjB1 enhances its ability to suppress α-synuclein aggregation.^13^

The association between clinical Parkinsonism and DNAjB2 variants has only been reported previously in 4 patients. One case carried a homozygous pathogenic variant c.145delG in DNAjB2, presented with typical Parkinson’s disease at the age of 50 years, with good response to levodopa.^3^ Another research discovered a large DNAjB2 deletion in a patient with spinal muscular atrophy and juvenile Parkinsonism with poor response to levodopa treatment.^12^

We describe the case of a 36-year-old female who presented with CMT and PD who later was found to have a DNAjB2 gene mutation. Written informed consent was obtained from the patient for this case report.

## Case Presentation

The patient, a 36-year-old woman, was initially evaluated at our neurology clinic at age 28 for a long-standing history of gait abnormalities and progressive bilateral lower extremity weakness, which she had first noticed during her teenage years. She has been followed longitudinally for 9 years (November 2016–June 2025), and over time, her symptoms gradually worsened, culminating in a bilateral foot drop, prompting a neurology referral. A detailed neurological examination revealed bilateral distal lower extremity weakness, most prominent in ankle dorsiflexion, absent deep tendon reflexes, impaired sensation to pain, temperature, and proprioception in a stocking distribution, and a high-steppage gait compensating for bilateral foot drop. The progression of her symptoms was gradual, with periods of stability followed by worsening, which is characteristic of both CMT2 and PD.

**Figure 1.**
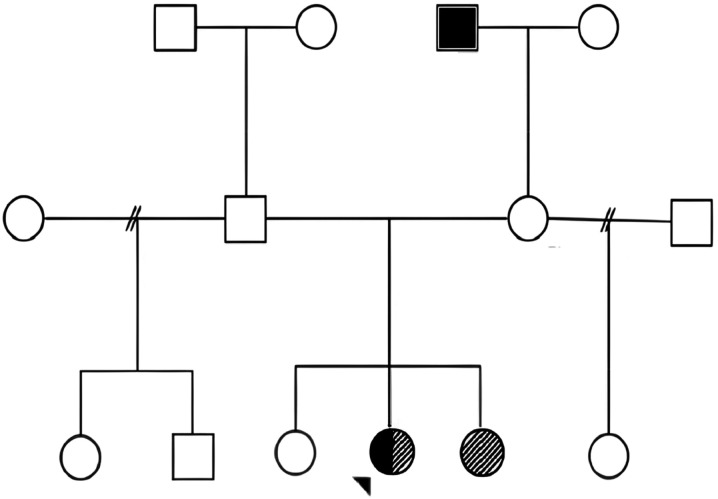
Pedigree of the reported family with co-existing Charcot–Marie–Tooth disease type 2 (CMT2) and Parkinson’s disease (PD) associated with a DNAJB2 variant

One of her first-degree relatives (sister) had a similar clinical presentation of distal extremity weakness and gait abnormalities, raising suspicion for an inherited condition in our patient (Figure 1).

The proband (arrow) has both CMT2 and PD. One full sister has CMT2 only, and another is unaffected. The maternal grandfather has PD. Other relatives, including parents, paternal grandparents, and half-siblings, are unaffected. The pattern suggests autosomal dominant inheritance with variable expressivity and familial segregation of the DNAJB2 variant.

**Legend Key:**

• **Solid black** - Parkinson’s disease (PD)
• **Diagonal stripes** - Charcot–Marie–Tooth disease type 2 (CMT2)
• **Half solid/half striped** - Both PD and CMT2
• **Empty-** Unaffected

Electrodiagnostic studies, including electromyography (EMG) and nerve conduction studies (NCS), demonstrated a severe axonal sensorimotor polyneuropathy, characterized by a marked reduction in both motor and sensory amplitudes, particularly in the lower limbs. These findings supported a diagnosis of hereditary axonal neuropathy, specifically Charcot-Marie-Tooth disease type 2 (CMT2).

**Figure 2.**
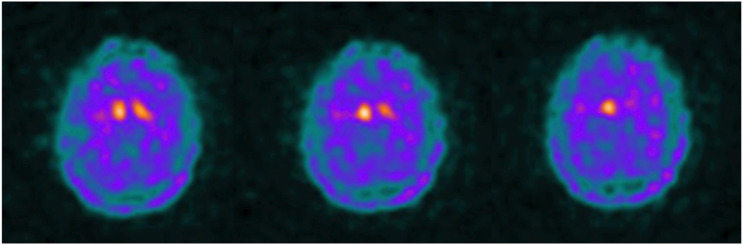
DaTscan imaging showing decreased radiotracer uptake in the bilateral striatum

At age 34, the patient developed a unilateral rest tremor of the right hand. A follow-up neurological examination showed right-side rest tremors, bradykinesia, and rigidity, leading to the diagnosis of young-onset Parkinsonian syndrome. A DaTscan (Dopamine transporter SPECT imaging) revealed a significant decrease in uptake in both striatum (Figure 2). Her motor symptoms responded well to a trial of carbidopa/levodopa. Although her PD symptoms gradually progressed to include tremors, rigidity, and bradykinesia on the opposite side, she continued to walk independently. Notably, the patient’s maternal grandfather was diagnosed with Parkinson’s disease in his early 50s, suggesting possible familial PD.

To investigate a possible genetic etiology underlying both phenotypes, the patient underwent comprehensive genetic testing, including a complete hereditary neuropathy and Parkinsonism panel. The hereditary neuropathy panel identified two pathogenic variants in the DNAjB2 gene: c.352+1 G>A and c.175+2T>A. Both are splice-site variants that disrupt normal splicing, resulting in the loss of functional protein. This biallelic variant pattern is consistent with autosomal recessive CMT2 due to dysfunction of DNAjB2. The Parkinson’s disease gene panel identified no pathogenic variants or known risk-associated variants. Unfortunately, further confirmatory genetic data from other family members could not be obtained due to financial constraints and insurance-related barriers that prevented them from accessing genetic testing.

## Discussion

Here, we presented the dual phenotype of CMT2 and young-onset PD in a patient with pathogenic DNAjB2 variants. The identified c.352 + 1G > A and c.175 + 2T > A variants are splice site variants that are likely to disrupt the standard processing of DNAjB2 mRNA, leading to truncated or non-functional protein forms. Evidence from previously published case reports is limited, and the proposed pathogenic mechanism of the DNAjB2 variants in the causation of Parkinson’s disease and CMT2 remains speculative.^6-8,12,14,15^ The pathogenic variant of c.352 + 1G > A is more commonly found in patients presenting with CMT. Homozygotic variants of c.352 + 1G > A were found in a patient who developed typical PD at the age of 50 years with asymmetric rest tremor, rigidity, and hypokinesia responsive to levodopa treatment. The mobility of that case is profoundly impaired by severe weakness.^3^ A large deletion of DNAJB2 was found in an autosomal recessive consanguineous family presenting with SMA and juvenile Parkinsonism. One patient from that family presented with coarse resting tremors and rigidity on the right side at the age of 16 with a moderate response to levodopa therapy.^12^ c.175 + 2T > A variants have not been reported in association with either CMT or PD. Our case developed PD symptoms at age 34 with a rather good response to Levodopa therapy, and she remained ambulating independently.

Young-onset Parkinson’s Disease (YOPD) is usually indicated in patients who develop PD symptoms before age 40. Several genetic mutations, such as PARK2, PINK1, DJ-1, and LRRK2, have been linked to YOPD. With the addition of our case report, the DNAjB2 gene and other DNAJ protein family should be considered possible causative genes in such cases, especially when concurrently presenting with peripheral neuropathy.

The DNAjB2 gene is vital in maintaining protein homeostasis by preventing the aggregation of misfolded proteins and facilitating their degradation via the ubiquitin-proteasome and autophagy pathways. This insight can guide further genetic therapies aimed at upregulating DNAjB2 activity and mitigating the pathological accumulations of misfolded proteins.^5,15^ A recent study found that EGFR-mediated phosphorylation of DNAjB1 plays a crucial role in the clearance of α-synuclein, and it may provide insights into the DNAjB protein as a potential therapeutic target.^13^

## Conclusion

Dual neuronal dysfunction in coexistent axonal CMT and young-onset Parkinson’s disease underscores a broader role for the DNAjB2 gene in neurodegeneration in both the peripheral and central nervous systems. The unique and novel compound heterozygous pathogenic variants in DNAjB2 may explain the complexity of the clinical phenotypes in our case.

## References

[1] Timmerman V Strickland AV Züchner S . Genetics of Charcot-Marie-Tooth (CMT) disease within the frame of the Human Genome Project success. Genes. 2014;5(1):13-32. doi:10.3390/genes5010013. 24705285 PMC3978509

[2] Sarparanta J Jonson PH Reimann J , et al. Extension of the DNAJB2a isoform in a dominant neuromyopathy family. Hum Mol Genet. 2023;32(21):3029-3039. doi:10.1093/hmg/ddad058. 37070754 PMC10586202

[3] Saveri P Magri S Maderna E , et al. DNAJB2-related Charcot-Marie-Tooth disease type 2: Pathomechanism insights and phenotypic spectrum widening. Eur J Neurol. 2022;29(7):2056-2065. doi:10.1111/ene.15326. 35286755 PMC9314055

[4] Zhang K Pan H Zhao Y , et al. Genetic analysis of HSP40/DNAJ family genes in Parkinson’s disease: A large case-control study. Mol Neurobiol. 2022;59(9):5443-5451. doi:10.1007/s12035-022-02920-5. 35715682

[5] McKinnon C De Snoo ML Gondard E , et al. Early-onset impairment of the ubiquitin-proteasome system in dopaminergic neurons caused by α-synuclein. Acta neuropathol commun. 2020;8:17. doi:10.1186/s40478-020-0894-0. 32059750 PMC7023783

[6] Clausen L Okarmus J Voutsinos V Meyer M Lindorff-Larsen K Hartmann-Petersen R . PRKN-linked familial Parkinson's disease: cellular and molecular mechanisms of disease-linked variants. Cell Mol Life Sci. 2024;81(1):223. doi:10.1007/s00018-024-05262-8. 38767677 PMC11106057

[7] Frasquet M Rojas-García R Argente-Escrig H , et al. Distal hereditary motor neuropathies: Mutation spectrum and genotype-phenotype correlation. Eur J Neurol. 2021;28(4):1334-1343. doi:10.1111/ene.14700. Epub 2021 Jan 10. Erratum in: Eur J Neurol. 2022 Mar;29(3):955. doi: 10.1111/ene.15244. 33369814

[8] Frasquet M Chumillas MJ Vílchez JJ , et al. Phenotype and natural history of inherited neuropathies caused by HSJ1 c.352+1G>A mutation. J Neurol Neurosurg Psychiatry. 2016;87(11):1265-1268. doi:10.1136/jnnp-2015-312890. 27083531

[9] Gess B Auer-Grumbach M Schirmacher A , et al. HSJ1-related hereditary neuropathies: novel mutations and extended clinical spectrum. Neurology. 2014;83(19):1726-1732. doi:10.1212/WNL.0000000000000966. 25274842

[10] Lazzarini E Jongbloed JD Pilichou K , et al. The ARVD/C genetic variants database: 2014 update. Hum Mutat. 2015;36(4):403-410. doi:10.1002/humu.22765. 25676813

[11] Teive H Kok F Raskin S Arruda W . Distal hereditary motor neuropathy with HSJ1 chaperone mutation, presenting with peripheral motor neuropathy, associated to parkinsonism, and cerebellar ataxia: case report. Parkinsonism Related Disord. 2015;22:e154.

[12] Sanchez E Darvish H Mesias R , et al. Identification of a large DNAJB2 deletion in a family with spinal muscular atrophy and Parkinsonism. Hum Mutat. 2016;37(11):1180-1189. doi:10.1002/humu.23055. 27449489 PMC5375037

[13] Huang YY Lin SJ Chiang WY , et al. EGFR phosphorylates DNAJB1 to suppress α-synuclein aggregation in Parkinson’s disease. npj Parkinsons Dis. 2025;11:157. doi:10.1038/s41531-025-01006-y. 40483356 PMC12145416

[14] He Y Wang Z . The roles of the HSP40/DNAJ protein family in neurodegenerative diseases. Zhejiang Da Xue Xue Bao Yi Xue Ban. 2022;51(5):640-646. doi:10.3724/zdxbyxb-2021-0406. 36581576 PMC10264987

[15] Zarouchlioti C Parfitt DA Li W Gittings LM Cheetham ME . DNAJ proteins in neurodegeneration: essential and protective factors. Philos Trans R Soc Lond B Biol Sci. 2018;373(1738):20160534. doi:10.1098/rstb.2016.0534. 29203718 PMC5717533

